# Development of PCR Methods for Detecting Wheat and Maize Allergens in Food

**DOI:** 10.3390/biotech14040078

**Published:** 2025-10-01

**Authors:** Tata Ninidze, Tamar Koberidze, Kakha Bitskinashvili, Tamara Kutateladze, Boris Vishnepolsky, Nelly Datukishvili

**Affiliations:** 1 School of Natural Sciences and Medicine, Ilia State University, 3/5 Kakutsa Cholokashvili Ave., Tbilisi 0162, Georgia; tata.ninidze.1@iliauni.edu.ge (T.N.); tamar.koberidze.3@iliauni.edu.ge (T.K.); 2 Ivane Beritashvili Center of Experimental Biomedicine, 14 Gotua Str., Tbilisi 0160, Georgia; kakha.bitskinashvili.1@iliauni.edu.ge (K.B.); kutateladzet@yahoo.com (T.K.); b.vishnepolsky@lifescience.org.ge (B.V.)

**Keywords:** wheat glutenin, maize allergens, Zea m 14, Zea m 8, zein, allergen detection, PCR

## Abstract

The detection of allergens is essential for ensuring food safety, protecting public health, and providing accurate information to consumers. Wheat (*Triticum aestivum* L.) and maize (*Zea mays* L.) are recognized as important food allergens. In this study, novel PCR methods were developed for the reliable detection of wheat and maize allergens, including wheat high-molecular-weight glutenin subunit (HMW-GS) and low-molecular-weight glutenin subunit (LMW-GS), as well as three maize allergens, namely, Zea m 14, Zea m 8, and zein. Wheat and maize genomic DNA, as well as allergen genes, were examined during 60 min of baking at 180 °C and 220 °C. Agarose gel electrophoresis revealed degradation of genomic DNA and amplified PCR fragments in correlation with increasing baking temperature and time. For each target gene, the best primers were identified that could detect HMW-GS and LMW-GS genes in wheat samples and Zea m 14, Zea m 8, and zein genes in maize samples after baking at 220 °C for 60 min and 40 min, respectively. The results indicate that these PCR methods can be used for the reliable and sensitive detection of wheat and maize allergens in processed foods.

## 1. Introduction

Wheat (*Triticum aestivum* L.) and maize (*Zea mays* L.) are staple food crops that play a crucial role in the global food industry. They are among the most cultivated and high-yielding important cereals in the world. Wheat and maize (corn), together with rice, account for about 90% of total cereal production and provide about 50% of the food calories for the world’s population [1,2,3,4]. Wheat and maize are important sources of carbohydrates, proteins, vitamins, minerals, dietary fiber, and antioxidants. They are widely used as raw materials and ingredients in the preparation of local food products in various countries. Wheat bread and pasta, maize porridge and oil, as well as their cereals, flours, tortillas, and various types of baked goods are considered essential foods for human nutrition worldwide [5,6]. However, wheat and maize foods can cause allergic reactions in sensitive individuals. Due to the widespread use of wheat and maize products, research into their allergenicity is particularly important.

Food allergy is recognized worldwide as one of the major challenges to food safety and public health. According to available data, 2–10% of the world’s population has a food allergy, and the number of sufferers is increasing globally every year. A food allergy develops when the immune system reacts negatively to a specific food. It can cause a variety of reactions that can be severe and sometimes life-threatening, such as anaphylaxis [7,8,9]. At present, no universally accepted treatment exists for food allergies. Accordingly, strict avoidance of the harmful foods remains the sole established method for disease prevention. The effective management of food allergies depends on the availability of precise and accurate allergen information for consumers. To ensure public health, international and domestic legislations have been developed and implemented that require mandatory labeling of allergenic foods. Therefore, the reliable detection of allergens is crucial for accurate food labeling and safety assessment, as well as for consumer information and health protection [10,11,12,13].

Specific proteins in certain foods trigger allergic reactions. The characterization and reliable detection of allergenic proteins and their encoding genes allows for the assessment of the allergenicity of foods. Correspondingly, allergen detection is carried out by two main approaches based on protein or DNA, including various tools such as liquid chromatography, mass spectrometry, gel electrophoresis, blotting, polymerase chain reaction (PCR), biosensors, etc. [14,15,16]. Among them, two methods are most widely used, namely, protein-based enzyme-linked immunosorbent assay (ELISA) and DNA-based PCR.

Each of them has its own specifications, advantages and limitations, which are summarized and discussed in detail for specific allergens and food matrices [17]. Recent studies have shown that the allergenicity of foods can be reduced, increased, or not changed at all by processing [18]. Due to the greater stability of DNA over proteins during food processing, PCR technology provides a reliable and sensitive method for detecting food ingredients [14,19].

Available data show that food matrix composition and processing significantly affect genomic DNA quality and quantity, limiting the applicability of PCR [20]. Studies on plant-based matrices demonstrate that technological treatments (i.e., temperature, pH, pressure) reduce DNA integrity and impair PCR detection. For reliable analysis of processed foods, target amplicons should be limited to ~200–300 bp [21,22]. Recent studies indicate that food processing influences allergenic proteins and their encoding genes [23,24]. Therefore, studying allergen detectability during processing is essential for accurately assessing the allergenicity of processed food products.

Wheat ranks among the major allergenic food crops and is extensively cultivated worldwide [25]. Wheat and products derived from it can cause various allergic reactions, including celiac disease, wheat sensitivity, and allergy, which can lead to fatal anaphylaxis in genetically susceptible individuals. Wheat-related disorders are globally prevalent, affecting approximately 7% of the population [26,27]. Therefore, wheat allergy is one of the significant challenges for global food safety and public health.

The primary cause of wheat allergenicity is its high level of gluten proteins, such as glutenin and gliadins, which are present in similar amounts and together make up about 80–85% of wheat protein. Gluten proteins are essential for key properties of wheat, including its adaptability, productivity, and the unique elasticity of its dough. However, these proteins also trigger allergic reactions [28,29,30]. A lifelong wheat-exclusion diet remains the only effective treatment for wheat-related disorders. Gluten detection methods play a crucial role in ensuring a safe diet for patients with wheat allergies. Several techniques have been developed for the detection and quantification of gluten, including ELISA and mass spectrometry [31,32,33], as well as PCR [34,35,36,37,38,39]. The advantages of the PCR method include good sensitivity, specificity, and avoidance of false-positive results.

Maize can elicit allergic reactions not only through ingestion but also via inhalation of its flour or dust [40,41,42]. Due to the widespread use of foods made from maize, preventing corn allergies is becoming increasingly difficult. There are 20 different types of corn allergen proteins, among which the lipid transfer proteins (LTPs- Zea m 14), profilin (Zea m 12), chitinase (Zea m 8), and zein stand out [43,44,45,46]. To date, only a limited number of studies have explored the detection of maize allergens using liquid chromatography, mass spectrometry, and polymerase chain reaction (PCR) approaches [47,48,49,50].

Despite these efforts, significant gaps remain in our understanding of how various food processing conditions affect the integrity and amplifiability of DNA. Furthermore, due to the limitations of current analytical techniques, selecting the most suitable method for detecting specific allergens in processed food products remains challenging. Therefore, new studies on the impact of different food processing conditions on allergen assessment are of particular interest.

This study aimed to develop efficient PCR methods for detecting wheat and maize allergens in processed food. For this purpose, the amplification of major allergen genes, namely, wheat HMW glutenin and LMW glutenin as well as maize Zea m 14, Zea m 8, and zein, was investigated with newly designed PCR primers. Additionally, the effect of high-temperature baking on the integrity of wheat and maize DNA, with a particular focus on the detectability of the allergen-encoding genes, was evaluated.

## 2. Materials and Methods

### 2.1. Plant Material and Sample Preparation

Wheat (*Triticum aestivum*) and maize (*Zea mays*) food products, such as wheat flour, maize flour, wheat bread, wheat and maize crispbread, pasta, canned sweet corn, oat cookie, salty sticks, oatmeal, buckwheat crispbread, instant noodles, potato chips, cereal breakfast, crunchy puffcorn snack, tortilla chips, round chips, sweet puffcorn, crunchy puffcorn snack, and chicken bouillon cube were purchased from local supermarkets in Tbilisi, Georgia. The samples for the baking were prepared as follows: 200 g of flour (separately for maize and wheat) was mixed with 200 g of drinking water and kneaded to a homogeneous dough. Maize and wheat dough samples weighing 50 g were baked in a preheated oven at 180 °C and 220 °C for 10, 20, 30, 40, 50, and 60 min. In each experiment, dough samples were also taken without heat treatment and dried at 35–40 °C. To enhance the genomic DNA extraction result, all dried dough samples were ground at 5000 rpm for 2 min using an electric grinder, Tube Mill 100 control (IKA, Staufen, Germany), to a flour-like consistency. While wheat and maize flours were used directly without any further processing.

### 2.2. DNA Extraction

DNA extraction was performed from 100 mg of each sample using the cetyltrimethyl ammonium bromide (CTAB)-based method as previously described [51]. The method included the following main steps. The sample was incubated with CTAB buffer and proteinase K at 65 °C, followed by RNase A treatment. After chloroform extraction, DNA was precipitated with CTAB solution, re-dissolved in NaCl, and re-extracted with chloroform. DNA was then precipitated with isopropanol, washed with 70% ethanol, air-dried, and, finally, dissolved in 100 μL sterile deionized water. The extracted DNAs were assessed through agarose gel electrophoresis, using 7 μL aliquots from each sample analyzed on a 1% agarose gel. In addition, DNA concentration and purity were estimated using NanoDrop One Microvolume UV-Vis Spectrophotometer (Thermo Fisher Scientific, Waltham, MA, USA) by measuring the UV absorbance at 260 nm (A260) and the ratios of the absorbance A260/A280 and A260/A230, respectively.

### 2.3. Bioinformatic Analysis and Design of Oligonucleotide Primers

For identification of wheat and maize allergens and their coding gene sequences, scientific literature, protein and allergen databases (https://www.uniprot.org, accessed on 10 January 2024; http://www.allergome.org, https://fermi.utmb.edu/SDAP/, accessed on 25 February 2024), and GenBank (https://www.ncbi.nlm.nih.gov/nucleotide, accessed on 17 November 2023) were screened. We selected two essential wheat allergens and identified their gene sequences, such as wheat Glu-1Ax1 gene for high-molecular-weight glutenin subunit 1Ax1 and *T. aestivum* low-molecular-weight glutenin storage protein mRNA. In addition, the genes coding three maize allergenic proteins, namely, *Z. mays* chitinase (chiA—Zea m 8), *Z. mays* subsp. *parviglumis* isolate p8 phospholipid transfer protein 1 (plt1—Zea m 14), and *Z. mays* 10 kDa zein, were selected.

The different oligonucleotide primers targeting above-mentioned genes were designed using Primer-BLAST [52] and PrimerQuest tools (https://eu.idtdna.com/PrimerQuest, accessed on 12 April 2024). The FastPCR-version 3.6.18 was used to evaluate the possible formation of dimers and secondary structures [53]. In addition, primer pairs targeting the eukaryotic 18S ribosomal RNA gene and chloroplast genome conserved region were taken from previous publications [54,55]. All of the oligonucleotides were synthesized by Integrated DNA Technologies (Coralville, IA, USA). The PCR primers used in this study are shown in Table 1.

### 2.4. PCR Analysis

PCR analyses were performed on the thermal cycler Techne TC-412 (Techne, Minneapolis, MN, USA). The amplification conditions for newly designed primer pairs were optimized by testing several parameters, including MgCl_2_ concentration (1.0–6 mM), primer concentration (0.1–1.5 μM), annealing temperature (52–68 °C), elongation time (20–90 s), and cycle number (30–60). After optimization, the PCRs were carried out in a final volume of 25 μL using 1.25 U Taq DNA polymerase, standard Taq Buffer, 1.5 mM MgCl_2_, 0.2 mM dNTP (New England BioLabs, Ipswich, MA, USA), 50–70 ng of genomic DNA, and 0.5 μM of each primer. The PCR cycling profiles are shown in Table 2.

The amplified products were detected by electrophoresis on 2.0% agarose gels; afterwards, they were evaluated under ultraviolet (UV) light and photographed using a gel documentation system PhotoDoc—It imaging system.

Each DNA extraction and PCR analysis was performed in at least duplicate across three independent experiments, and outcome comparisons confirmed the reproducibility of the results.

### 2.5. Statistical Analysis

Based on the spectrophotometric data (A260/A280, DNA concentration), results of DNA yield (ng DNA/fresh sample) were calculated, processed, and statistically analyzed using SigmaPlot software, Version 15.0 (Systat Software Inc., San Jose, CA, USA). A significance level of *p* < 0.05 was considered statistically significant.

## 3. Results

### 3.1. The Impact of Baking on the Wheat and Maize Genomic DNAs

Visual and textural differences between the samples have already been evident during kneading and baking. Specifically, the wheat dough demonstrated high elasticity and thinness, in contrast to the maize dough, which appeared denser and thicker in consistency. The high elasticity of wheat dough is due to the presence of gluten proteins in wheat flour, while maize flour is naturally gluten-free. The maize dough was baked quickly for about 30 min at 180 °C and 20 min at 220 °C. Subsequently, the maize bread began to darken progressively with increased baking time, becoming visibly charred after 50 min at 180 °C and 40 min at 220 °C. The wheat dough was baked on the outside for about 50 min at 180 °C and 60 min at 220 °C but remained moist on the inside. The wheat bread also browned, but later and with less intensity. Textural differences between wheat and maize samples influenced DNA integrity, as evidenced by subsequent DNA analysis and PCR amplification results.

**Figure 1:**
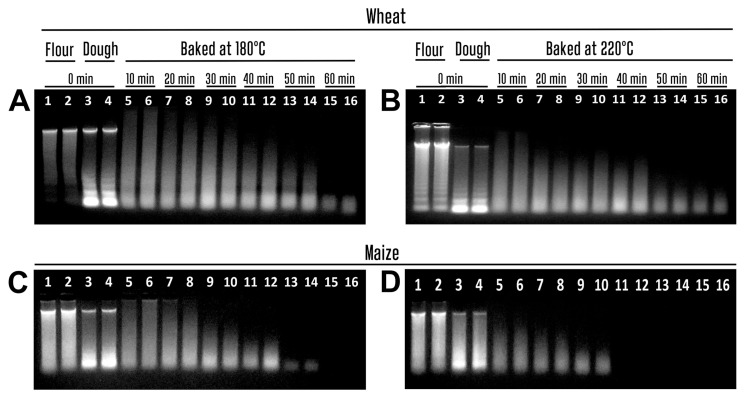
Genomic DNAs from wheat (**A**,**B**) and maize (**C**,**D**) samples treated at 180 °C ((**A**,**C**) lanes 5–16) and 220 °C ((**B**,**D**) lanes 5–16). Baking time: 0 min ((**A**–**D**) lanes 1–4); 10 min ((**A**–**D**) lanes 5–6); 20 min ((**A**–**D**) lanes 7–8); 30 min ((**A**–**D**) lanes 9–10); 40 min ((**A**–**D**) lanes 11–12); 50 min ((**A**–**D**) lanes 13–14); 60 min ((**A**,**B**) lanes 15–16). Samples: wheat flour ((**A**,**B**) lanes 1–2); maize flour ((**C**,**D**) lanes 1–2); wheat dough ((**A**,**B**) lanes 3–16); maize dough ((**C**,**D**) lanes 3–16).

Figure 1 shows an agarose gel electrophoresis of the genomic DNAs from wheat (Figure 1A,B) and maize (Figure 1C,D) samples taken during 60 min of baking at 180 °C and 220 °C. Comparison of DNA electrophoretic images indicates both time and temperature dependent degradation of both maize and wheat genomes. The influence of 220 °C on DNA integrity was significantly stronger than that of 180 °C. The intensive bands of high-molecular-weight DNA appeared in the thermally untreated samples of wheat flour (Figure 1A,B, lanes 1–2), maize flour (Figure 1C,D lanes 1–2), wheat dough (Figure 1A,B, lanes 3–4), and maize dough (Figure 1C,D, lanes 3–4); however, the dough DNAs showed more smearing than the flour DNAs. The high-molecular-weight genomic DNA bands disappeared even after 10 min of baking, while DNA extracts from the baked samples contained strong smeared DNA bands. Moreover, the size of DNA fragments was reduced by the time and temperature of processing (Figure 1, lanes 5–16). Notably, different degrees of degradation were observed for maize and wheat genomic DNAs. In particular, the short DNA fragments were seen in wheat samples even after 60 min of baking, while no DNA bands appeared in maize samples after 60 min of baking at 180 °C and 40 min of baking at 200 °C.

Table 3 and Table 4 present the concentration/yield and purity values for the DNAs obtained from wheat and maize samples. It should be noted that since DNA was extracted from 100 mg of food and the final volume of DNA solution was 100 μL, the DNA concentration indicator (ng DNA/μL solution) exactly corresponds to the ratio of total DNA yield (ng DNA) per mg of food.

Comparative analysis of DNA yields revealed that baking time had a significant effect on DNA yield, whereas baking temperature exhibited no substantial impact. In addition, wheat samples yielded relatively more DNA than maize samples. Also, flour yielded the highest amount of DNA compared to dough samples (average 154.12 ng/mg wheat sample and 81.59 ng/mg maize sample). After kneading the dough, DNA yields decreased slightly (average 136.00 ng/mg wheat sample and 62.84 ng/mg corn sample). After baking, DNA yields decreased with increasing exposure time. The lowest DNA yields were obtained from baked samples after 60 min of treatment (average 34.27 ng/mg wheat sample and 2.01 ng/mg maize sample). The DNA yield was particularly sharply reduced (by 97%) for the maize sample, compared to 77% for the wheat sample.

The values of the absorbance A260/A280 and A260/A230 were used to estimate the purity of DNAs and the presence of organic contaminants, such as proteins, phenols, and other aromatic compounds. All of the wheat DNAs and most of the maize DNAs showed A260/A280 ratios greater than 1.8 and ratios A260/A230 greater than 1.9, indicating the absence of protein, phenolic, and other contamination in these DNA samples. Only four maize samples baked at 180 °C for 60 min and at 220 °C for 40, 50, and 60 min showed A260/A280 ≤ 1.7, and A260/A230 lower than 1.5, suggesting the presence of proteins, phenols, carbohydrates, and other contaminants in these samples. The results can be explained by the fact that during the baking of maize bread, the final samples were charred, which induced the formation of phenolic compounds, acrylamide, and other thermal processing contaminants.

**Figure 2:**
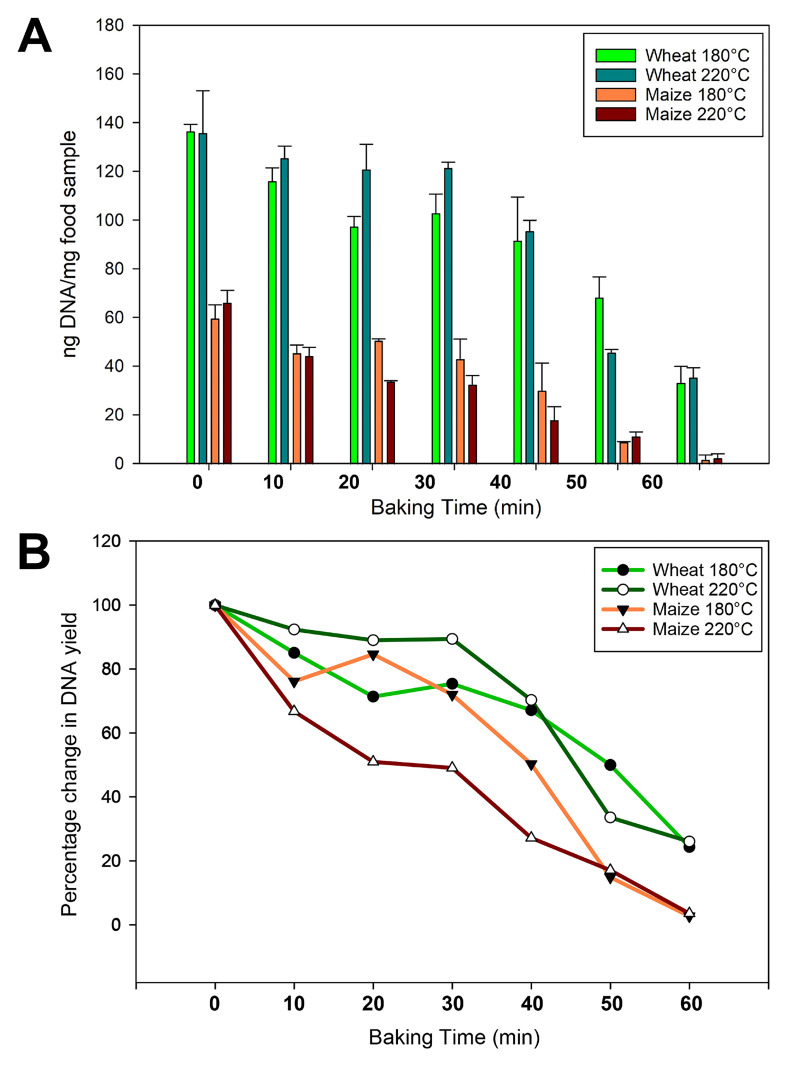
Spectrophotometric analysis of DNAs extracted from wheat and maize samples baked at 180 °C and 220 °C for 0, 10, 20, 30, 40, 50, and 60 min. (**A**)—effect of baking on the DNA yield; (**B**)—percentage change in DNA yield.

The effect of baking on the amount of extracted DNA in both wheat and maize samples is presented together in Figure 2. The extractability of DNAs mainly decreased with the time of baking. However, there were exceptions during the first 30 min of processing, namely, there was an increase in extracted DNA twice (samples at 180 °C, maize for 20 min and wheat for 30 min) and, also, the same amount of DNA was observed twice (maize and wheat at 180 °C for 30 min). We suggest that the similar or improved extractability observed in some cases may be attributed to the initial partial breakdown of high-molecular-weight DNA early in the baking process, which was then followed by more extensive degradation after 60 min of baking. It is noteworthy that such a result is typical for DNAs obtained by the CTAB method, which was also observed in early studies on DNA degradation in thermally processed foods [22,56].

### 3.2. Influence of Baking on the PCR Amplification of Wheat and Maize DNA

**Figure 3:**
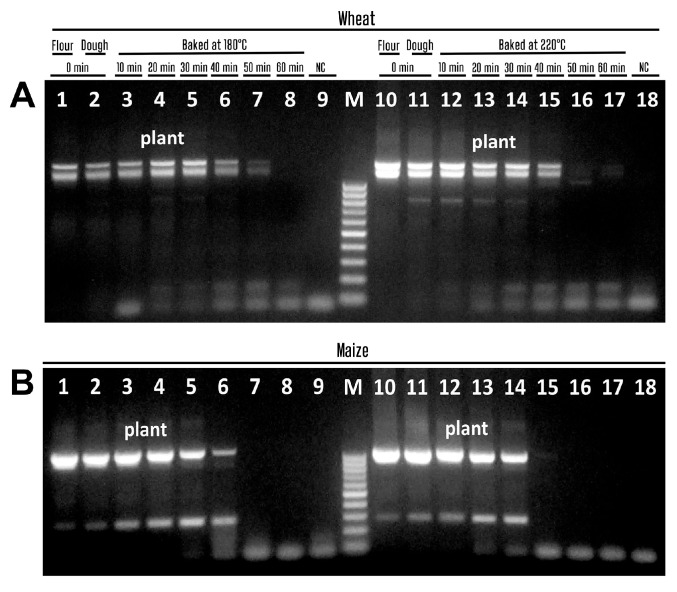
PCR amplification with primers plant1/plant2 of wheat (**A**) and maize (**B**) samples treated at 180 °C ((**A**,**B**) lanes 3–8) and 220 °C ((**A**,**B**) lanes 12–17). Baking time: 0 min ((**A**,**B**) lanes 1–2, 10–11, 9, 18); 10 min ((**A**,**B**) lanes 3, 12); 20 min ((**A**,**B**) lanes 4, 13); 30 min ((**A**,**B**) lanes 5, 14); 40 min ((**A**,**B**) lanes 6, 15); 50 min ((**A**,**B**) lanes 7, 16); 60 min ((**A**,**B**) lanes 8, 17). Samples: wheat flour ((**A**) lanes 1, 10); maize flour ((**B**) lanes 1, 10); wheat dough ((**A**) lanes 2–8, 11–17); maize dough ((**B**) lanes 2–8, 11–17); Water-NC-Negative control ((**A**,**B**) lanes 9, 18). M. Molecular weight marker: Qiagen GelPilot 50 bp ladder.

To evaluate and compare the amplification efficacy of both wheat and maize genomes, we selected the primers plant1/plant2 and 18S-167f/18S-167r, which correspond to common plant sequences. The plant1 and plant2 primers target conserved sequences of the chloroplast genome and produce main amplicons ranging from approximately 500 to 700 base pairs in size, depending on the plant species [55]. Figure 3 presents the results of agarose gel electrophoresis of the PCR products generated using the plant1/plant2 primers. Both wheat (Figure 3A) and maize (Figure 3B) samples were treated at temperatures of 180 °C (lanes 3–8) and 220 °C (lanes 12–17) for investigation.

The electrophoretic images showed expected PCR fragments in the range of 500–700 bp for both maize and wheat DNA templates (Figure 3). However, the wheat samples gave two bands about 600–700 bp in size (Figure 3A, lanes 1–7; lanes 10–15) while maize samples generated one main amplicon about 500–600 bp in size and a second PCR fragment about 150 bp in size (Figure 3B, lanes 1–6, lanes 10–14). In addition, amplified products completely disappeared in wheat samples after 60 min of baking at 180 °C (Figure 3A, lane 8) and after 50 min of baking at 180 °C (Figure 3A, lanes 16–17), as well as in maize samples after 50 min of baking at 180 °C (Figure 3B, lanes 7, 8) and after 40 min of baking at 180 °C (Figure 3B, lanes 15–17).

**Figure 4:**
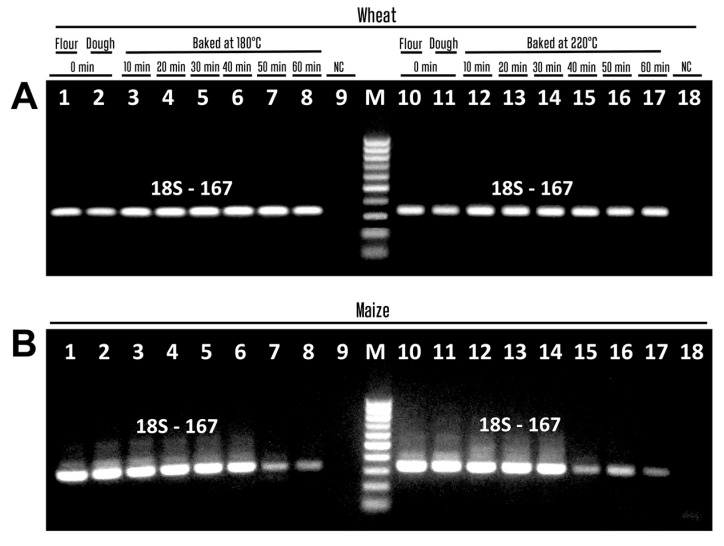
PCR amplification with primers 18S-167f/18S-167r of wheat (**A**) and maize (**B**) samples treated at 180 °C ((**A**,**B**) lanes 3–8) and 220 °C ((**A**,**B**) lanes 12–17). Baking time: 0 min ((**A**,**B**) lanes 1–2, 10–11, 9, 18); 10 min ((**A**,**B**) lanes 3, 12); 20 min ((**A**,**B**) lanes 4, 13); 30 min ((**A**,**B**) lanes 5, 14); 40 min ((**A**,**B**) lanes 6, 15); 50 min ((**A**,**B**) lanes 7, 16); 60 min ((**A**,**B**) lanes 8, 17). Samples: wheat flour ((**A**) lanes 1, 10); maize flour ((**B**) lanes 1, 10); wheat dough ((**A**) lanes 2–8, 11–17); maize dough ((**B**) lanes 2–8, 11–17); Water-NC-Negative control ((**A**,**B**) lanes 9, 18). M. Molecular weight markers: Qiagen GelPilot 50 bp ladder.

Figure 4 shows PCR products amplified with primers 18S-167f/18S-167r targeting the eukaryotic 18S ribosomal RNA gene. The expected 167 bp amplicons were generated in all samples of wheat (Figure 4A, lanes 1–8, 10–17) and maize (Figure 4B, lanes 1–8, 10–17). In addition, no amplified products were seen in negative water controls (Figure 3A,B, lanes 9, 18; Figure 4A,B, lanes 9, 18), suggesting the purity of the PCR experiments. The results show that the extracted DNA samples do not contain PCR inhibitors. It was also found that for successful PCR, it is important that the amplicon length is less than 500 bp.

### 3.3. Impact of Baking on the PCR Detection of Wheat Allergens

**Figure 5:**
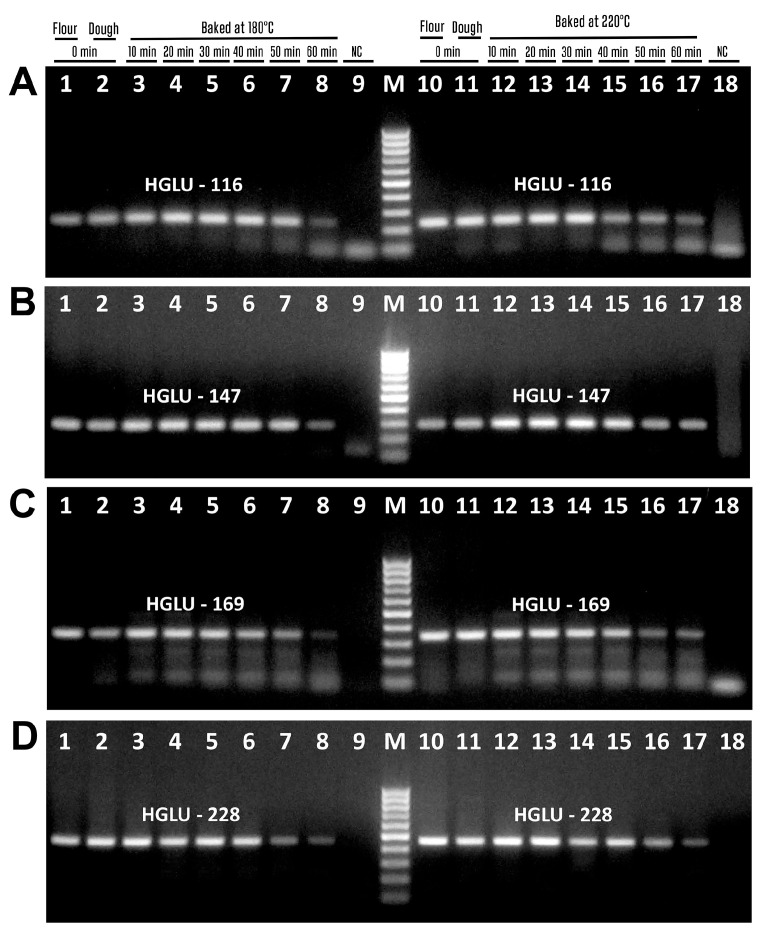
PCR amplification with primers Hglu-116f/Hglu-116r (**A**), Hglu-147f/Hglu-147r (**B**), Hglu-169f/Hglu-169r (**C**), Hglu-228f/Hglu-228r (**D**) of wheat (**A**) samples treated at 180 °C ((**A**–**D**) lanes 3–8) and 220 °C ((**A**–**D**) lanes 12–17). Baking time: 0 min ((**A**–**D**) lanes 1–2, 10–11, 9, 18); 10 min ((**A**–**D**) lanes 3, 12); 20 min ((**A**–**D**) lanes 4, 13); 30 min ((**A**–**D**) lanes 5, 14); 40 min ((**A**–**D**) lanes 6, 15); 50 min ((**A**–**D**) lanes 7, 16); 60 min ((**A**–**D**) lanes 8, 17). Samples: wheat flour ((**A**–**D**) lanes 1, 10); wheat dough ((**A**–**D**) lanes 2–8, 11–17); Water-NC-Negative control ((**A**–**D**) lanes 9, 18). M. Molecular weight markers: Qiagen GelPilot 50 bp ladder.

Figure 5 presents PCR products of wheat samples amplified with four pairs of primers corresponding high-molecular-weight glutenin subunit (Wheat HMW-GS) gene. The amplicons of expected size were generated for all samples of wheat DNAs, i.e., the primers Hglu-116f/Hglu-116r generated 116 bp amplicon (Figure 5A, lanes 1–8, 10–17), the primers Hglu-147f/Hglu-147r produced 147 bp amplicon (Figure 5B, lanes 1–8, 10–17), the primers Hglu-169f/Hglu-169r produced 169 bp amplicon (Figure 5C, lanes 1–8, 10–17), and Hglu-228f/Hglu-228r primers generated 228 bp products (Figure 5D, lanes 1–8, 10–17).

**Figure 6:**
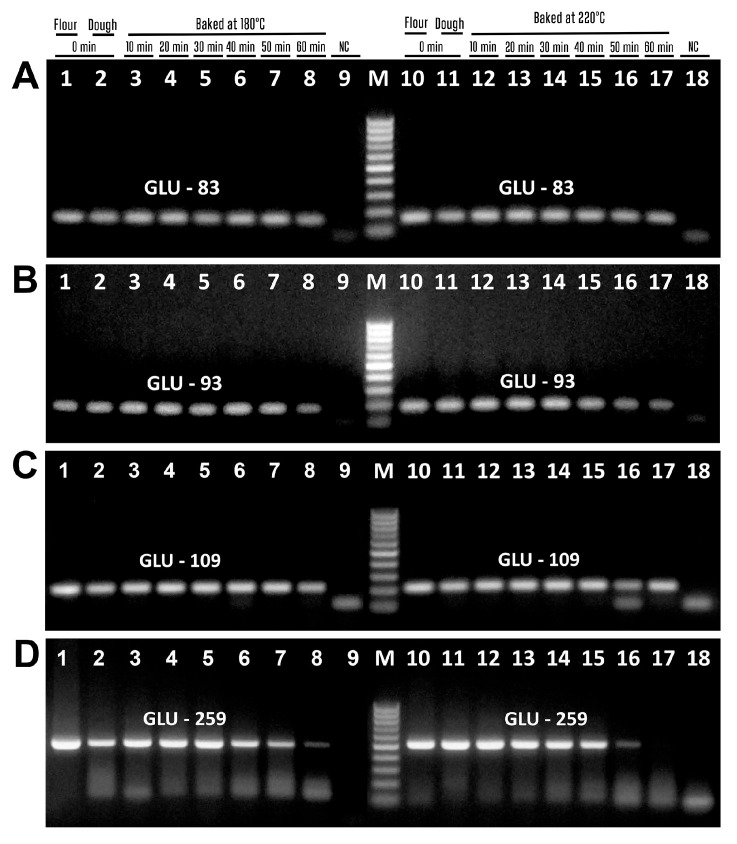
PCR amplification with primers Glu-83f/Glu-83r (**A**), Glu-93f/Glu-93r (**B**), Glu-109f/Glu-109r (**C**), Glu-259f/Glu-259r (**D**) of wheat samples treated at 180 °C ((**A**–**D**) lanes 3–8) and 220 °C ((**A**–**D**) lanes 12–17). Baking time: 0 min ((**A**–**D**) lanes 1–2, 10–11, 9, 18); 10 min ((**A**–**D**) lanes 3, 12); 20 min ((**A**–**D**) lanes 4, 13); 30 min ((**A**–**D**) lanes 5, 14); 40 min ((**A**–**D**) lanes 6, 15); 50 min ((**A**–**D**) lanes 7, 16); 60 min ((**A**–**D**) lanes 8, 17). Samples: wheat flour ((**A**–**D**) lanes 1, 10); wheat dough ((**A**–**D**) lanes 2–8, 11–17); Water-NC-Negative control ((**A**–**D**) lanes 9, 18). M. Molecular weight markers: Qiagen GelPilot 50 bp ladder.

The amplified products of wheat samples produced with four pairs of primers targeting low-molecular-weight glutenin subunit (Wheat LMW-GS) are shown in Figure 6. Each PCR reaction produced the suitable amplicon, i.e., the primers Glu-83f/Glu-83r produced 83 bp amplicon (Figure 6A, lanes 1–8, 10–17), Glu-93f/Glu-93r primers produced 93 bp amplicon (Figure 6B, lanes 1–8, 10–17), Glu-109f/Glu-109r primers generated 109 bp product (Figure 6C, lanes 1–8, 10–17), and Glu-259f/Glu-259r primers generated 259 bp product Figure 6D, lanes 1–8, 10–16).

However, GLU-259 amplicons disappeared after baking at 220 °C for 60 min (Figure 6D, lane 17). No amplified products were seen in water samples (Figure 5A–D, lanes 9, 18; Figure 6A–D, lanes 9, 18), indicating the purity and specificity of the PCR experiments.

### 3.4. Impact of Baking on the PCR Detection of Maize Allergens

**Figure 7:**
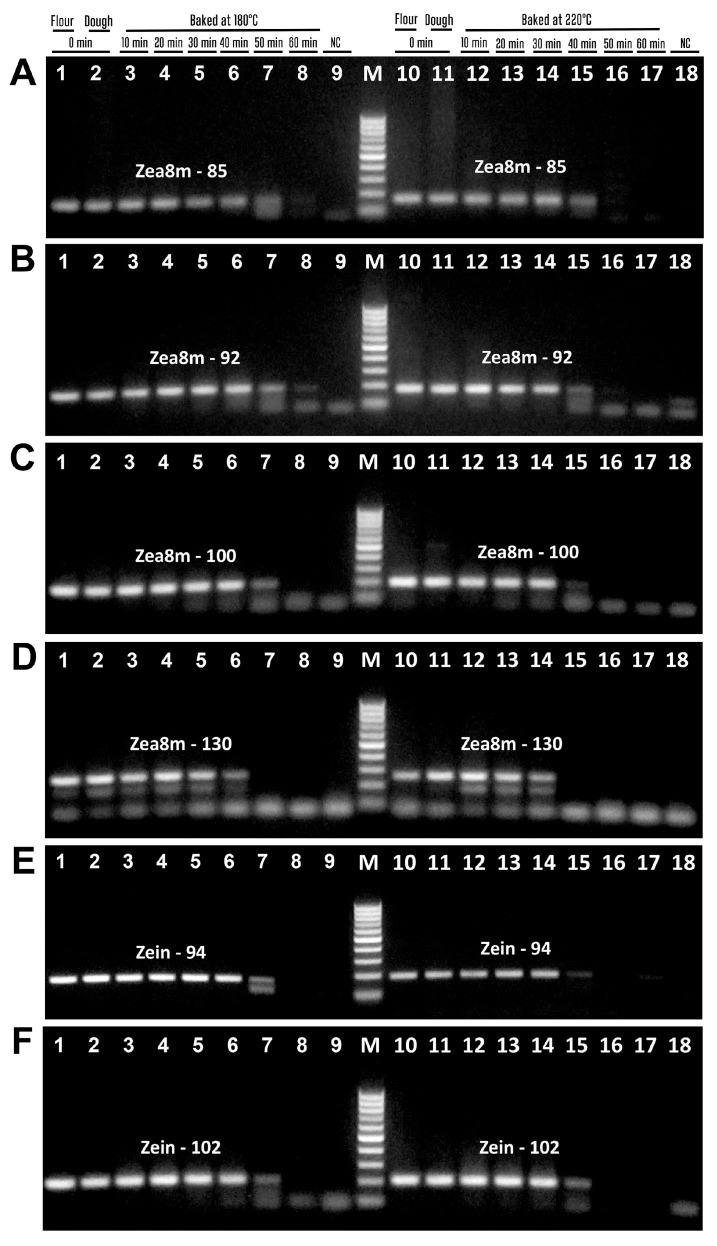
PCR amplification with primers zea8m85f/zea8m85r (**A**), zea8m92f/zea8m92r (**B**), zea8m-100f/zea8m-100r (**C**), zea8m-130f/zea8m-130r (**D**), zein-94f/zein-94f zein-94r (**E**), zein102f/zein102r (**F**) of maize samples treated at 180 °C ((**A**–**F**) lanes 3–8) and 220 °C ((**A**–**F**) lanes 12–17). Baking time: 0 min ((**A**–**F**) lanes 1–2, 10–11, 9, 18); 10 min ((**A**–**F**) lanes 3, 12); 20 min ((**A**–**F**) lanes 4, 13); 30 min ((**A**–**F**) lanes 5, 14); 40 min ((**A**–**F**) lanes 6, 15); 50 min ((**A**–**F**) lanes 7, 16); 60 min ((**A**–**F**) lanes 8, 17). Samples: maize flour ((**A**–**F**) lanes 1, 10); maize dough ((**A**–**F**) lanes 2–8, 11–17); Water-NC-Negative control ((**A**–**F**) lanes 9, 18). M. Molecular weight markers: Qiagen GelPilot 50 bp ladder.

Four pairs of primers targeting the maize chitinase (Zea m 8) gene produced expected amplicons, i.e., zea8m85f/zea8m85r primers generated 85 bp product (Figure 7A, lanes 1–7, 10–15), zea8m92f/zea8m92r primers generated 92 bp product (Figure 7B, lanes 1–8, 10–15), zea8m-100f/zea8m-100r primers produced 100 bp amplicon (Figure 7C, lanes 1–7, 10–15), and zea8m-130f/zea8m-130r primers produced 130 bp amplicon (Figure 7D, lanes 1–6, 10–14). However, Zea8m-85 and Zea8m-92 amplicons disappeared after baking at 220 °C for 50 min (Figure 7A,B, lane 16). The bands of amplicon Zea8m-100 were absent after baking at 180 °C for 60 min (Figure 7C, lane 8) and after baking at 220 °C for 50 min (Figure 7C, lane 16). In addition, the bands of amplicon Zea8m-130 were absent after baking at 180 °C for 50 min (Figure 7D, lane 7) and after baking at 220 °C for 40 min (Figure 7C, lane 15).

Two pairs of primers targeting maize zein gene produced suitable PCR products, i.e., the primers zein-94f/zein-94f zein-94r generated 94 bp amplicon (Figure 7E, lanes 1–7, 10–15), and the primers zein102f/zein102r generated 102 bp amplicon (Figure 7F, lanes 1–7, 10–15). However, the bands of these amplicons were absent after baking at 180 °C for 60 min (Figure 7E, lane 8) and at 220 °C for 50 min (Figure 7E, lane 16).

**Figure 8:**
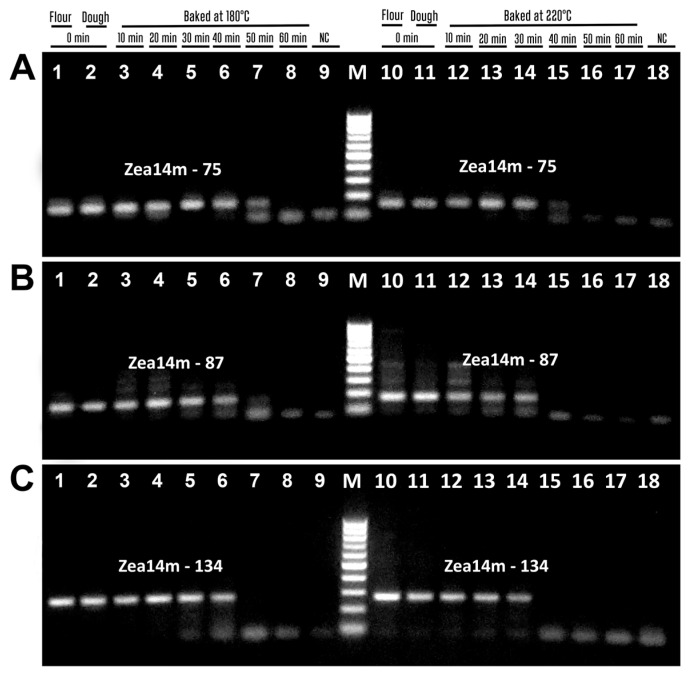
PCR amplification with primers zea14m-75f/zea14m-75r (**A**), zea14m87f/zea14m87r (**B**), zea14m134f/zea14m134r (**C**) of maize samples treated at 180 °C ((**A**–**C**) lanes 3–8) and 220 °C ((**A**–**C**) lanes 12–17). Baking time: 0 min ((**A**–**C**) lanes 1–2, 10–11, 9, 18); 10 min ((**A**–**C**) lanes 3, 12); 20 min ((**A**–**C**) lanes 4, 13); 30 min ((**A**–**C**) lanes 5, 14); 40 min ((**A**–**C**) lanes 6, 15); 50 min ((**A**–**C**) lanes 7, 16); 60 min ((**A**–**C**) lanes 8, 17). Samples: maize flour ((**A**–**C**) lanes 1, 10); maize dough ((**A**–**C**) lanes 2–8, 11–17); Water-NC-Negative control ((**A**–**C**) lanes 9, 18). M. Molecular weight markers: Qiagen GelPilot 50 bp ladder.

Figure 8 presents the amplification of the phospholipid transfer protein 1 (Zea m 14) gene using three pairs of suitable primers. The amplicons of expected size were produced, i.e., zea14m-75f/zea14m-75r primers generated 75 bp amplicon (Figure 8A, lanes 1–7, 10–15), zea14m87f/zea14m87r primers generated 87 bp amplicon (Figure 8B, lanes 1–7, 10–14), and zea14m134f/zea14m134r primers produced 134 bp amplicon (Figure 8C, lanes 1–6, 10–14). However, during baking at 180 °C, the amplicons Zea14m-75 and Zea14m-87 were no longer detectable after 60 min (Figure 8A,B, lane 8), whereas the Zea14m-134 amplicon was undetectable even after 50 min of treatment. In addition, during baking at 220 °C, the amplicons Zea14m-75 disappeared after 50 min (Figure 8A, lanes 16–17), while the amplicons Zea14m-87 and Zea14m-134 were absent even after 40 min (Figure 8B,C, lanes 15–17). It should be noted that no PCR products were formed in the negative water control in any of the PCR experiments, which indicates the high purity and specificity of the experiments (Figure 7 and Figure 8, lanes 9, 18).

The results of the amplification of all PCR products examined during baking are given in Table 5. Amplified amplicons can be seen for all genes of wheat allergen, but some of the PCR fragments of the maize allergens have not been amplified after 40 min of baking, and the negative result of PCR increased according to the continuation of processing. Moreover, all the amplicons of maize allergen genes were absent after 60 min of baking, except Zea8m92.

### 3.5. PCR Detection of Wheat and Maize Allergens in Food Products

**Figure 9:**
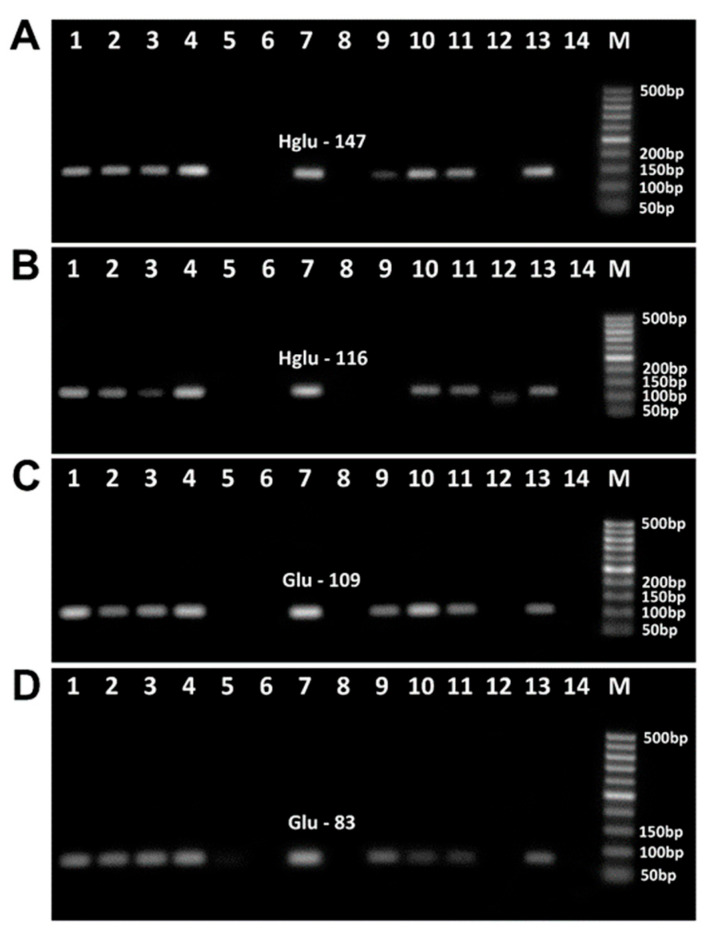
PCR detection of wheat allergens in foods with primers Hglu-147f/Hglu-147r (**A**); Hglu-116f/Hglu-116r (**B**), glu-109f/glu-109r (**C**), glu-83f/glu-83r (**D**). Samples: wheat flour ((**A**–**D**) lane 1); wheat bread ((**A**–**D**) lane 2); oat cookie ((**A**–**D**) lane 3); salty sticks ((**A**–**D**) lane 4); oatmeal ((**A**–**D**) lane 5); buckwheat crispbread ((**A**–**D**) lane 6); instant noodles ((**A**–**D**) lane 7); chicken bouillon cube ((**A**–**D**) lane 8); wheat and maize crispbread ((**A**–**D**) lane 9); potato chips ((**A**–**D**) lane 10); cereal breakfast ((**A**–**D**) lane 11); crunchy puffcorn snack ((**A**–**D**) lane 12); pasta ((**A**–**D**) lane 13); Water-NC-Negative control ((**A**–**D**) lane 14). M. Molecular weight markers: Qiagen GelPilot 50 bp ladder.

Figure 9 shows the analysis of wheat allergens in food products using primers selected from previous experiments on baked wheat bread. Two primer pairs, Hglu-147f/Hglu-147r (Figure 9A) and Hglu-116f/Hglu-116r (Figure 9B), targeted the HMW-GS gene, while glu-109f/glu-109r (Figure 9C) and glu-83f/glu-83r (Figure 9D) targeted the LMW-GS gene.

Amplicons of the expected size were detected in all food products derived from wheat or containing wheat ingredients, namely, wheat flour (Figure 9A–D, lane 1), wheat bread (Figure 9A–D, lane 2), salty sticks (Figure 9A–D, lane 4), instant noodles (Figure 9A–D, lane 7), wheat and maize crispbread (Figure 9A, C and D, lane 9), breakfast cereals (Figure 9A–D, lane 11), and pasta (Figure 9A–D, lane 13), except for the HMW-GS gene 116 bp fragment in crispbread (Figure 9B, lane 9). This indicates a lower detection sensitivity of Hglu-116f/Hglu-116r primers compared to other primers for the analysis of processed foods. In addition, all four PCR tests detected corresponding amplified bands in oat cookies (Figure 9A–D, lane 3) and potato chips (Figure 9A–D, lane 10), indicating the presence of a wheat ingredient in the suspect products. Finally, PCR products were not found in four suspect products, such as oatmeal (Figure 9A–D, line 5), buckwheat crispbread (Figure 9A–D, line 6), chicken bouillon cube (Figure 9A–D, line 8), and crunchy puffcorn snack (Figure 9A–D, line 12), indicating the absence of wheat ingredients in these foods.

**Figure 10:**
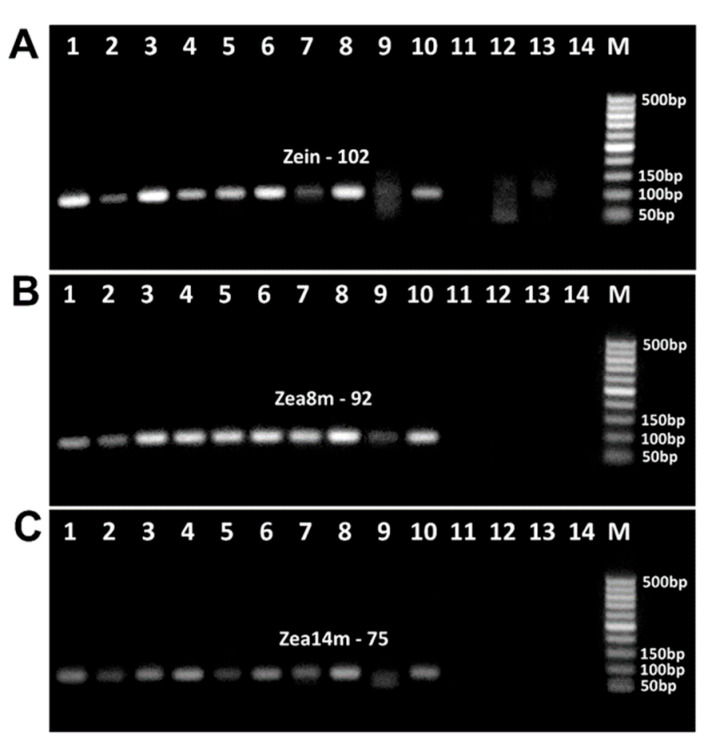
PCR detection of maize allergens in foods with primers zein102f/zein102r (**A**); zea8m92f/zea8m92r (**B**), zea14m75f/zea14m75r (**C**). Samples: maize flour ((**A**–**C**) lane 1); wheat and maize crispbread ((**A**–**C**) lane 2); canned sweet corn ((**A**–**C**) lane 3); tortilla chips ((**A**–**C**) lane 4); cereal breakfast ((**A**–**C**) lane 5); round chips ((**A**–**C**) lane 6); sweet puffcorn ((**A**–**C**) lane 7); crunchy puffcorn snack ((**A**–**C**) lane 8); buckwheat crispbread ((**A**–**C**) lane 9); potato chips ((**A**–**C**) lane 10); chicken bouillon cube ((**A**–**C**) lane 11); oat cookies ((**A**–**C**) lane 12); instant noodles ((**A**–**C**) lane 13); Water-NC-Negative control ((**A**–**C**) lane 14); M. Molecular weight markers: Qiagen GelPilot 50 bp ladder.

Figure 10 shows the analysis of maize allergens in food products by PCR with effective primers selected based on previous experiments on baked maize bread. Three primer pairs were used, including zein102f/zein102r targeting the zein gene (Figure 10A), zea8m92f/zea8m92r targeting the Zea m8 gene (Figure 10B), and zea14m75f/zea14m75r targeting the Zea m14 gene (Figure 10C).

Expected PCR amplicons were detected in all foods derived from maize or containing maize ingredients, such as maize flour (Figure 10A–C, line 1), wheat and maize crispbread (Figure 10A–C, line 2), canned sweet corn (Figure 10A–C, line 3), tortilla chips (Figure 10A–C, line 4), breakfast cereals (Figure 10A–C, line 5), round chips (Figure 10A–C, line 6), sweet puffcorn (Figure 10A–C, line 7), and crunchy puffcorn snacks (Figure 10A–C, lane 8). In addition, amplified products were found in two suspect foods, such as buckwheat crispbread (Figure 10A–C, line 9) and potato chips (Figure 10A–C, line 10). However, amplicons were not found in two suspect food products, namely, chicken bouillon cubes (Figure 10A–C, line 11) and oat cookies (Figure 10A–C, line 12), as well as in a wheat-derived product, instant noodles (Figure 10A–C, line 13), indicating the absence of maize ingredients in these foods.

The detection of specific amplicons in all expected processed wheat and maize samples, along with the absence of PCR products in water controls and negative food matrices, demonstrates the high purity, specificity, and sensitivity of the developed PCR methods.

## 4. Discussion

The accurate detection of food allergens is essential for ensuring food safety, protecting public health, enabling precise product labeling, and providing reliable information to consumers [15]. This paper describes novel PCR-based methods for detecting wheat and maize allergens. Furthermore, this study evaluated, for the first time, the effects of the baking process on the integrity of wheat and maize DNA, as well as the stability and detectability of allergen-encoding genes. Wheat and maize, despite being highly allergenic, are among the most important food crops worldwide due to their significant nutritional value and economic relevance [25,43].

This study used baking as one of the main traditional heat treatment methods commonly used in food production. In addition, bakery goods constitute a significant portion of wheat and maize food products. Recently, there has been an important increase in consumer interest in bakery products that may have health benefits through changes in bioactive compounds or allergenicity. Despite previous studies, there remains a gap in understanding how different baking conditions affect food DNA and its diagnostic outcomes [5,22,23].

To assess the allergenicity of baked goods, PCR was selected as the most accurate, specific, and widely used tool for food analysis [17,18,19]. The results of food PCR analysis depend on many factors, including the food matrix, the polyploidy and genome size of the species, the genetic locus and copy number of the target gene, the food processing regime and parameters, the PCR primers, and the fragment length [24,25,58]. To our knowledge, this study is the first to present a comparative characterization of maize and wheat genomes and allergen genes during the baking process.

In this study, various wheat and maize samples, such as flour, dough, laboratory-prepared baked goods, as well as real processed food products, were analyzed. They differ in their food matrix, which includes physical and chemical structure, components, and their interactions. To assess species-specific matrix effects, food samples were prepared by kneading wheat and maize doughs using equal quantities of flour (200 g) and water (200 mL). Additionally, the kneading, preparation, and baking of doughs made from wheat and maize flour were performed simultaneously under identical conditions by a single researcher to ensure procedural consistency. This made it possible to identify differences due to the plant species matrices based on the comparison of results.

Baking, as a thermal treatment approach, influences the yield of high-quality and sufficient quantities of genomic DNA, which is crucial for successful PCR detection [21,22]. In this work, genomic DNA was extracted using the CTAB method, based on previous findings on the suitability of this method for obtaining large amounts of amplifiable DNA from processed foods [56]. A significant difference in DNA yield was found between wheat and maize species. Approximately two times more genomic DNA was extracted from 100 mg of wheat flour and dough than from maize samples. Furthermore, the yield of both wheat and maize DNA decreased with increasing baking time. However, different rates of change were observed depending on the species, namely, wheat DNA yield decreased by approximately 50% after 50 min of baking at both 180 °C and 220 °C, while maize DNA yield decreased by approximately 50% after 40 min of baking at 180 °C and 30 min of baking at 220 °C. Furthermore, after baking at both 180 °C and 220 °C for 60 min, wheat samples retained approximately 25% of the initial DNA yield, while maize samples showed only 3.2% of the initial DNA yield. Thus, the results showed a higher extractability of wheat DNA than of maize DNA. This may be due to the different matrices of wheat and maize flours and doughs, since the sample processing conditions were identical.

Species effects were also observed on the degradation of both genomic DNA and PCR amplicons. The comparison of DNA electrophoretic images clearly indicated the degradation of both maize and wheat genomes depending on baking duration and temperature. However, maize DNA bands completely disappeared after 60 min of baking at 180 °C and after 40 min of baking at 220 °C, whereas wheat DNA bands remained present in all samples throughout the baking process. This may be due to the higher concentration of wheat DNA compared to maize.

Two PCR systems targeting multi-copy conserved regions of the plant genomes were used to compare the amplifiability of wheat and maize genomic DNA. Using these systems, the characteristics of DNA extracts, such as DNA degradation and quantity, and presence of PCR inhibitors in them, as well as the estimated sizes of amplicons for successful PCR, were checked. Significant differences were observed using the plant1/plant2 primers, namely, they generated two amplicons in wheat samples, with a size of approximately 600 bp–700 bp, while maize samples produced a single major amplicon of 500 bp. This is consistent with previous results on the production of chloroplast genome PCR products that are of various sizes in the range of 500–700 bp, depending on the plant species [51,54,55]. In addition, the maize amplicon degraded earlier and faster during baking than the wheat amplicons. As for the 167 bp amplicons of the 18S RNA gene, they were present in both wheat and maize samples throughout the entire 60 min of baking. However, their amount was significantly reduced in the heavily processed maize samples, while it remained almost the same in wheat. Our results confirmed earlier findings about the important role of food matrix and species specificity in DNA extractability and analytical assessment [20,22,58].

The reliable detection of genes encoding allergenic proteins to ensure food safety remains a significant challenge in molecular biotechnology. In this study, different PCR primers were investigated for the detection of important allergen genes, such as wheat HMW-GS and LMW-GS, as well as maize Zea m 14, Zea m 8, and zein. The results clearly showed a higher detectability of wheat amplicons than maize amplicons. It was found that all wheat amplicons were visible on the agarose gel throughout the baking period, except for Glu-259 after 60 min at 220 °C, while some maize amplicons disappeared in the heavily treated samples after 40, 50, or 60 min of baking. This can be explained by the significantly higher DNA concentration and higher number of gene copies in wheat samples compared to maize samples. In more detail, bread wheat has a hexaploid genome, while maize has a diploid one [59,60,61,62]. In addition, wheat allergens have multi-copy genes with high allelic diversity, while corn allergen genes are low-copy genes. Specifically, the wheat genome contains six copies of HMW-GS genes and about 15–30 copies of LMW-GS genes [28,29,63]. However, the maize genome contains one copy of the Zea m 8 gene and 2–3 copies of the 10-kDa zein gene. The specific copy number of the Zea m 14 gene is not clearly described in the available literature. It has been suggested that there may be multiple copies of Zea m 14 in the maize genome [43,64,65,66]. This study confirmed the importance of gene copy number and species polyploidy in the amplification efficiency of PCR fragments.

Given previous results on the advantage of short amplicons in PCR analysis of processed foods [21], amplicons shorter than 300 bp were generated using newly designed primers to detect allergen genes. The amplification results showed that most wheat primers were able to detect glutenin genes even in highly processed samples after baking at 220 °C for 60 min, while maize-specific primers were only able to detect allergen genes in moderately processed samples after baking at 220 °C for 30 or 40 min. In addition, the results identified efficient PCR methods for each allergen gene, which generate fragments of 147 bp and 116 bp for the HMW-GS gene and 83 bp and 109 bp for the LMW-GS gene, fragments of 92 bp for the Zea m 8 gene and 75 bp for the Zea m 14 gene, and fragments of 94 bp and 102 bp for the Zein gene.

The investigation of amplicons of various sizes and different genetic loci revealed a negative correlation between amplicon size and its detectability. It was demonstrated by PCR systems targeting plant-specific high-copy-number genes using primers plant1/plant2 and 18S-167f/18S-167r. While 500–700 bp amplicons of the chloroplast genome disappeared after 30–50 min of baking, 167 bp amplicons of the 18S RNA gene were still present in all wheat and maize samples throughout the entire 60 min of baking. Similar results were observed for allergen gene amplicons. In particular, after baking at 220 °C for 60 min, the longest wheat PCR fragment, 259 bp, was no longer visible, while other shorter fragments ranging from 83 bp to 228 bp were amplified. In addition, the longest maize PCR fragment, 134 bp, disappeared before the other shorter fragments.

Furthermore, it has been found that PCR results are influenced by the genetic location of the amplicons. In particular, a comparison of maize amplicons of similar size showed that fragments from the Zea m 14 gene degraded more rapidly than fragments from the Zea m 8 and zein genes. In addition, in some cases, long amplicons showed higher detectability than short amplicons of the same gene, which may be due to the higher efficiency of the PCR primers targeting the long amplicon. In particular, the 92 bp zea8m92 amplicon was detectable even after baking at 180 °C for 60 min, while shorter, 85 bp PCR fragments of the same Zea m 8 gene disappeared under these conditions. Thus, the results indicate that amplicon size, along with gene locus and primer efficiency, also affect PCR detection. It is noteworthy that the presence of 167 bp PCR fragments in all DNA samples indicates the presence of amplifiable genomic DNA in them. However, different results of other PCRs confirm the crucial role of various factors, such as species matrix and polyploidy, amplicon size, gene locus, and primer specificity, in allergen detectability.

Baking at 180 °C and 220 °C was chosen because these temperatures are widely applied in the production of baked goods. Samples were tested every 10 min during the 60 min baking period to determine the effect of processing temperature and duration on PCR detection. The results showed that genomic DNAs and their PCR fragments were degraded during baking, and the impact of 220 °C was significantly stronger than that of 180 °C. Moreover, DNA yield was greatly reduced upon baking, with only 3% of the initial amount remaining in the maize samples and 23% in the wheat samples after 60 min. However, baking temperature did not significantly affect DNA yield. The amount of each amplified product decreased with increasing processing time and temperature. However, the rate of change depended on the length and amount of the amplicon, as well as the genetic locus. The results obtained showed that the integrity and yield of genomic DNA, as well as amplicons, are affected by food cooking. However, the intensity of the impact is defined by the texture of the plant/food, cooking time, and duration. The outcomes of this study coincide with earlier findings on DNA and amplicon degradation depending on the time and temperature of heat treatment of food products [21,22,23,24].

Thus, the results obtained indicate that the PCR-based detection of processed products depends on a combination of various important factors, such as the ploidy and texture of the plant species, gene locus and copy number, amplicon size, the efficiency of PCR primers, food matrix, treatment temperature, and duration. This suggests that PCR assay conditions and parameters should be optimized for each food sample under a separate processing mode.

The study evaluated the suitability of newly developed PCR methods for detecting wheat and corn allergens in processed foods. A broad range of products was analyzed, including differently processed wheat and maize items (wheat flour, maize flour, wheat bread, wheat and maize crispbread, pasta, and canned sweet corn), mixed food matrices with varying ingredient compositions and wheat/maize contents (salty sticks, instant noodles, cereal breakfast, crunchy puffcorn snack, tortilla chips, round chips, and sweet puffcorn), and matrices in which wheat or maize were absent or of uncertain presence (oat cookie, oatmeal, buckwheat crispbread, potato chips, and chicken bouillon cube). The detection of allergens in highly processed, mixed-matrix wheat and corn foods confirms the effectiveness of the diagnostic methods. The results confirmed the high reliability, specificity, and sensitivity of these PCR methods for detecting wheat and maize allergens in processed foods.

## 5. Conclusions

For the first time, this study investigated how baking affects the integrity of wheat and maize DNA, along with the stability and detectability of their allergen-encoding genes. The outcomes of this study demonstrated that the sensitivity of PCR detection positively correlates with DNA extractability, species polyploidy, and gene copy number. However, PCR sensitivity negatively correlates with amplicon length, processing temperature, and duration. Additionally, the specificity of primers and the gene locus affect the results of PCR analysis. Therefore, this study provides crucial insights for food testing technologies. Moreover, this information will contribute to the improvement of DNA diagnostic approaches in various fields of biotechnology.

This study identified new PCR methods for wheat and maize specific allergens, the effectiveness of which has been confirmed by testing baked goods and various processed foods. This study identified gluten-specific PCR methods suitable for detecting allergen genes in highly processed wheat products after baking at 220 °C for 60 min. However, PCR methods targeting the 147 bp and 116 bp fragments of the HMW-GS gene and the 83 bp and 109 bp fragments of the LMW-GS gene were found to be most effective. In addition, maize-specific PCR methods have been developed that detect allergen genes in processed products after baking at 220 °C for 40 min, and the best sensitivity was shown with PCR methods generating 92 bp fragments of the Zea m 8 gene, 75 bp fragments of the Zea m 14 gene, and 102 bp fragments of the Zein gene. The results indicate that the developed polymerase chain reaction (PCR) methods are suitable for the reliable and accurate detection of wheat and corn allergens and can be successfully used in food safety assessment.

## Figures and Tables

**Table 1:** PCR oligonucleotide primers designed to specifically target sequences of *T. aestivum* (HMW glutenin, LMW glutenin), *Z. mays* (Zea m 8, Zea m 14, and zein), conserved regions of eukaryotic nuclear (18S rRNA), and chloroplast genome.

Primer	Sequence 5′→3′	Target Gene/ GenBank ID	Amplicon Size (bp)	Reference
Hglu-116f	AGGCTAAACAAACCTTACCGT	Wheat HMW-GS	116	This study
Hglu-116r	TTGTGATGCTCGGTGTTGTG	X61009.1		
Hglu-147f	TAGGCTAAACAAACCTTACCGTG	Wheat HMW-GS	147	This study
Hglu-147r	CGGTGGACTGTCGGTGAAT	X61009.1		
Hglu-169f	GGCATGCCGACAGGTCGTA	Wheat HMW-GS	169	This study
Hglu-169r	ACTTTGTTGGAGTTGCTGTGGT	X61009.1		
Hglu-228f	CAATGGCTGCAATCAGGGT	Wheat HMW-GS	228	This study
Hglu-228r	AACATGGTATGGGCTGTCGT	X61009.1		
Glu-83f	AATCCCGCTATGAGGCAATC	Wheat LMW-GS	83	This study
Glu-83f	AGATTGGATGGAACCCTGAAC	U86029.1		
Glu-93f	TTGCAGCCACACCAGATAG	Wheat LMW-GS	93	This study
Glu-93r	GTACAACGGCACATTAACACTG	U86029.1		
Glu-109f	GGTGGTTCCTGGGCTACTATAA	Wheat LMW-GS	109	This study
Glu-109r	GGAAGGTCTTCATGGTGGATTG	U86029.1		
Glu-259f	CAAGGTATTCCTCCAGCAGTGCAGC	Wheat LMW-GS	259	[56]
Glu-259r	GGGTTGGGAAACACATTGGCCCA	U86029.1		
zea14m-75f	GCCTCAACGCCGGTAAC	Maize Zea m 14	75	This study
zea14m-75f	TGGAGGTGCTGATGGTGTA	DQ147199.1		
zea14m87f	GCCTCAACGCCGGTAAC	Maize Zea m 14	87	This study
zea14m87r	TGGAGCAGTCGGTGGAG	DQ147199.1		
zea14m134f	CGCCCTGCATCTCCTAC	Maize Zea m 14	134	This study
zea14m134r	AGCGTTCTTGAGGCAGTT	DQ147199.1		
zea8m85f	TCAACGGCATCAAGAACCA	Maize Zea m 8	85	This study
zea8m85r	TTGTTGACGGCGCTCAG	GQ856537.1		
zea8m92f	AACGTGGCTAACGTGGTC	Maize Zea m 8	92	This study
zea8m92r	CTCCGGGTGTAGAAGTTCTTG	GQ856537.1		
zea8m-100f	GGTGCGAACGTGGCTAAC	Maize Zea m 8	100	This study
zea8m100r	CGCTCCGGGTGTAGAAGT	GQ856537.1		
zea8m-130f	GAACGTGGCTAACGTGGTCA	Maize Zea m 8	130	[50]
zea8m-130r	GAAGCCCGGGTACTTGTTGA	GQ856537.1		
zein-94f	GACGATTCCACCCATGTTCTTA	Maize zein	94	This study
zein-94r	CGATGGCATGTCAACTCATTATTC	U25674.1		
zein102f	ACACCACCGACCATGGCAGC	Maize zein	102	[57]
zein102r	TGGTGGCAAGTGCGCTGGAA	U25674.1		
18S-167f	GCAAGACCGAAACTCAAAGGA	18S rRNA	167	[54]
18S-167r	ACGACAGCCATGCAGCACC	X16077		
plant1	CGAAATCGGTAGACGCTACG	Chloroplast genome	500–600	[55]
plant2	GGGGATAGAGGGACTTGAAC	000932.1		

**Table 2:** Optimized PCR conditions for amplification of maize and wheat allergen genes, as well as conserved sequences of the 18S rRNA and chloroplast genome.

Target Gene	Cycles	Initial Denaturation	Denaturation	Annealing	Elongation	Final Extension
Chloroplast genome	35	95 °C for 4 min	95 °C for 30 s	62 °C for 30 s	72 °C for 2 min	72 °C for 5 min
18S rRNA	35	95 °C for 4 min	95 °C for 40 s	56 °C for 45 s	72 °C for 45 s	72 °C for 5 min
Maize Zein	40	95 °C for 3 min	95 °C for 30 s	65 °C for 30 s	72 °C for 35 s	72 °C for 3 min
Maize Zea m 8 Maize Zea m 14	40	95 °C for 3 min	95 °C for 30 s	60 °C for 30 s	72 °C for 35 s	72 °C for 5 min
Wheat LMW glutenin	35	94 °C 2 min	94 °C 30 s	60 °C 30 s	72 °C 1 min	72 °C 3 min
Wheat HMW glutenin	35	94 °C 3 min	95 °C 40 s	56 °C 45 s	72 °C 45 s	72 °C 5 min

**Table 3:** DNA concentration/yield and purity of the wheat samples baked at 180 °C and 220 °C.

	Samples	DNA Concentration (ng/μL) DNA Yield (ng/mg Food)	A260/A280	A260/A230
	Wheat flour	154.12 ± 15.63	1.88 ± 0.00	2.189 ± 0.01
	Dough	136.30 ± 3.00	1.88 ± 0.01	2.27 ± 0.01
Baked at 180 °C	10 min	115.95 ± 5.54	1.89 ± 0.01	2.09 ± 0.08
	20 min	97.34 ± 0.64	1.88 ± 0.01	2.05 ± 0.07
	30 min	102.83 ± 7.84	1.87 ± 0.02	2.18 ± 0.00
	40 min	91.53 ± 17.93	1.87 ± 0.01	2.08 ± 0.01
	50 min	68.21 ± 8.36	1.85 ± 0.01	2.05 ± 0.00
	60 min	33.195 ± 6.71	1.84 ± 0.03	1.90 ± 0.25
	Dough	135.69 ± 17.37	1.88 ± 0.01	2.26 ± 0.02
Baked at 220 °C	10 min	125.35 ± 4.98	1.89 ± 0.00	2.20 ± 0.04
	20 min	120.80 ± 10.34	1.89 ± 0.01	2.19 ± 0.01
	30 min	121.36 ± 2.46	1.91 ± 0.01	2.15 ± 0.03
	40 min	95.43 ± 4.52	1.89 ± 0.00	2.09 ± 0.06
	50 min	45.58 ± 1.30	1.85 ± 0.00	1.93 ± 0.01
	60 min	35.34 ± 3.95	1.87 ± 0.01	1.91 ± 0.03

Mean and standard deviation were calculated for the data obtained from DNA concentration and purity. Values were then expressed as Mean ± SD.

**Table 4:** DNA concentration/yield and purity of the maize samples baked at 180 °C and 220 °C.

	Samples	DNA Concentration (ng/μL) DNA Yield (ng/mg Food)	A260/A280	A260/A230
	Maize flour	81.59 ± 1.90	1.82 ± 0.04	2.46 ± 0.25
	Dough	59.61 ± 5.58	1.80 ± 0.02	2.57 ± 0.16
Baked at 180 °C	10 min	45.40 ± 3.21	1.88 ± 0.13	2.38 ± 0.42
	20 min	50.46 ± 0.64	1.88 ± 0.02	2.51 ± 0.27
	30 min	42.93 ± 8.11	1.84 ± 0.03	2.94 ± 0.22
	40 min	30.00 ± 11.18	1.89 ± 0.09	2.56 ± 0.19
	50 min	8.89 ± 0.10	1.85 ± 0.05	3.95 ± 2.90
	60 min	1.65 ± 1.80	1.33 ± 0.97	1.43 ± 2.19
	Dough	66.06 ± 5.01	1.80 ± 0.03	2.94 ± 0.23
Baked at 220 °C	10 min	44.13 ± 3.58	1.88 ± 0.02	2.77 ± 0.63
	20 min	33.69 ± 0.24	1.81 ± 0.07	3.36 ± 0.06
	30 min	32.45 ± 3.65	1.82 ± 0.03	2.81 ± 0.19
	40 min	18.00 ± 5.35	1.20 ± 0.16	0.61 ± 0.02
	50 min	11.29 ± 1.63	1.29 ± 0.09	0.65 ± 0.01
	60 min	2.36 ± 1.57	1.14 ± 0.27	0.46 ± 0.05

Mean and standard deviation were calculated for the data obtained from DNA concentration and purity. Values were then expressed as Mean ± SD.

**Table 5:** DNA amplification during baking of wheat and maize samples.

Target Gene	Amplicon Name	Amplicon Size (bp)	Baking Temp (°C)	Baking Time (min)						
				0	10	20	30	40	50	60
Wheat HMW-GS	HGLU-116	116	180 °C	+	+	+	+	+	+	+
			220 °C	+	+	+	+	+	+	+
	Hglu-147	147	180 °C	+	+	+	+	+	+	+
			200 °C	+	+	+	+	+	+	+
	Hglu-169	169	180 °C	+	+	+	+	+	+	+
			200 °C	+	+	+	+	+	+	+
	Hglu-228	228	180 °C	+	+	+	+	+	+	+
			200 °C	+	+	+	+	+	+	+
Wheat LMW-GS	Glu-83	83	180 °C	+	+	+	+	+	+	+
			200 °C	+	+	+	+	+	+	+
	Glu-93	93	180 °C	+	+	+	+	+	+	+
			220 °C	+	+	+	+	+	+	+
	Glu-109f	109	180 °C	+	+	+	+	+	+	+
			220 °C	+	+	+	+	+	+	+
	Glu-259f	259	180 °C	+	+	+	+	+	+	+
			220 °C	+	+	+	+	+	+	-
Maize Zea m 14	zea14m-75	75	180 °C	+	+	+	+	+	+	-
			220 °C	+	+	+	+	+	-	-
	zea14m87	87	180 °C	+	+	+	+	+	-	-
			220 °C	+	+	+	+	-	-	-
	zea14m134	134	180 °C	+	+	+	+	-	-	-
			220 °C	+	+	+	-	-	-	-
Maize Zea m 8	zea8m85	85	180 °C	+	+	+	+	+	+	-
			220 °C	+	+	+	+	+	-	-
	zea8m92f	92	180 °C	+	+	+	+	+	+	+
			220 °C	+	+	+	+	+	-	-
	zea8m-100f	100	180 °C	+	+	+	+	+	+	-
			220 °C	+	+	+	+	+	-	-
	zea8m-130	130	180 °C	+	+	+	+	+	-	-
			220 °C	+	+	+	+	-	-	-
Maize zein	zein-94f	94	180 °C	+	+	+	+	+	+	-
			220 °C	+	+	+	+	+	-	-
	zein102f	102	180 °C	+	+	+	+	+	+	-
			220 °C	+	+	+	+	+	-	-
18S rRNA	Maize and wheat 18S-167f	167	180 °C	+	+	+	+	+	+	+
			220 °C	+	+	+	+	+	+	+
Chloroplast genome	Maize plant	500	180 °C	+	+	+	+	+	-	-
			220 °C	+	+	+	+	-	-	-
Chloroplast genome	Wheat plant	600–700	180 °C	+	+	+	+	+	+	-
			220 °C	+	+	+	+	+	-	-

+ presence of amplicon, - absence of amplicon.

## Data Availability

All data generated and analyzed during this study are included in the present article.
